# Evaluating Cervicovaginal Infections and Cervical Cancer in Women with Low Socioeconomic Levels

**Published:** 2017-06

**Authors:** Soheila MORADI, Mehdi TADRIS HASANI, Leili DARVISH, Nasibeh ROOZBEH

**Affiliations:** 1. Mother and Child Welfare Research Center, Hormozgan University of Medical Sciences, Bandar Abbas, Iran; 2. Dept. of Psychology, Bandar Abbas Branch, Islamic Azad University, Bandar Abbas, Iran

## Dear Editor-in-Chief

Cervicovaginal infections are one of the most common problems in clinical medicine and 5 to 10 million women worldwide are diagnosed with this condition annually. Almost 95% of vaginitis cases are created with one of the three organisms including *Candida albicans, Gardenella vaginalis* and *Trichomonas vaginalis*, and they appear with symptoms such as vaginal discharge, itching, abnormal odor and vaginal discomfort. Although these infections are not life threatening, however, because of complications in patients, a waste of time and high costs of treatment had a particular importance (1). Normal cervical tissue and cancer cells may vulnerable to grow under the influence of infectious agents such as bacteria, fungi, viruses and parasites, normal cervical tissue and cancer cells. One of the viruses associated with cervical cancer is the human papillomavirus (2).

The incidence of cancer in different regions of the world is in terms of sexual behavior and different socioeconomic conditions (3). Most of these cases are seen in developing countries and poor families. Several factors have been mentioned for cervical cancer, including illiteracy, low education, low social and economic status, etc. (4). Cervical cancer is one of the most common cancers among females (5). This study evaluated cervicovaginal infections and cervical cancer in women with low socioeconomic level (covered by the Relief Committee) in Bandar Abbas in 2015. According to the relief committee’s request, 350 women under the Relief Committee at the University of Medicine were studied using Pap Smear sample of infection and cervical cancer.

After filling out the consent form, the demographic questionnaire was completed for them and these samples were studied. Pap Smear sample was prepared by two experienced midwives (with a history of over 15 yr) using standard techniques. Most of people in the studied population were in the age group of 36–50 yr (53.3%). Most of women in the study were homemakers (95.2%). The education level in this population (61.9%) was often primary and secondary level. About 57.1% of them married at the age of 18–35 yr and most of them between the ages of 18–35 yr had experienced their first pregnancy. 57.8% of them experienced 1–4 pregnancies, 40% of them had five or more past pregnancies, most of them had not a history of smoking and tobacco (91.9%) and no history of cervical cancer in their family. Fifty-nine percent of them had no previous history of genital tract infection and 69.6% of them had not already done a Pap Smear test. This study evaluated the samples of benign cellular changes, 230 number of them (66%) were infected with *Candida albicans*, 52 number of them (23%) only infected to this infection and 174 number of them (76%) had *Candida* with other infection. Overall, 308 number of women that surveyed (88%) were with bacterial vaginosis infection and 68 patients (19.5%) with *T. vaginalis*, however, 27 patients (7.8%) with herpes simplex virus (HSV). In this study, 47 patients (13.4%) were with invasive intraepithelial lesions type of ASCUS. Inflammatory cells were reported in 93% of prepared slides that was mild inflammation (10.4%), moderate (37.1%), and severe (45.5%). According to evaluating cervicovaginal cell cytology in the study population, there was no case of human papillomavirus.

The results of this study show many women with low socioeconomic level have experienced cervicovaginal infections and sexually transmitted infections like trichomoniasis as well as herpes simplex. In this study, 13.4% of women with low socioeconomic level had pre-invasive lesions intraepithelial. The relationship between low socioeconomic level of women and cervical cancer has been proven in other studies (6, 7). Due to the review articles summarizes the results of researches (8) can be useful to identify of the risk factors of cervical cancer in women with low socioeconomic levels. According to the high prevalence of cervicovaginal infections and invasive intraepithelial lesions in women with low socioeconomic level and since the women with low socioeconomic class compare the other women are more at risk, emphasized the use of proper and simple teaching methods for raising awareness in this group of women from cervical cancer screening and prevention methods.
